# Toll-Like Receptor 7 Stimulates the Expression of Epstein-Barr Virus Latent Membrane Protein 1

**DOI:** 10.1371/journal.pone.0043317

**Published:** 2012-08-31

**Authors:** Robert M. Valente, Erica Ehlers, Dongsheng Xu, Humera Ahmad, Andrew Steadman, Laura Blasnitz, You Zhou, Lisa Kastanek, Bin Meng, Luwen Zhang

**Affiliations:** 1 Arthritis Center of Nebraska, Lincoln, Nebraska, United States of America; 2 School of Biological Sciences, University of Nebraska, Lincoln, Nebraska, United States of America; 3 Nebraska Center for Virology, University of Nebraska, Lincoln, Nebraska, United States of America; 4 Center for Biotechnology, University of Nebraska, Lincoln, Nebraska, United States of America; 5 Department of Pathology, Shandong University School of Medicine, Jinan, Shandong, People's Republic of China; University of Hong Kong, Hong Kong

## Abstract

Epstein-Barr virus (EBV) is a ubiquitous human herpesvirus. Toll-like receptor 7 (TLR7) is involved in host innate immunity against pathogens, and its aberrant activation is linked to the development of systemic lupus erythematosus (SLE, also called “lupus”). Type I interferons (IFN) are apparently driving forces for lupus pathogenesis. Previously, we found that EBV latent membrane protein 1 (LMP1) primes cells for IFN production. In this report, the relationship among EBV LMP1, TLRs, and IFN production are examined. We find that TLR7 activation increases the expression of EBV LMP1, and IFN regulatory factor 7 (IRF7) is involved in the stimulation process. TLR7 activation did not induce IFNs from EBV-infected cells, but potentiates those cells for IFN production by TLR3 or TLR9 activation. In addition, we find that LMP1 and IFNs are co-expressed in the same cells in some lupus patients. Therefore, the aberrant activation of TLR7 might induce LMP1 expression and LMP1-expression cells may be producing IFNs in lupus patients. These results suggest EBV might be an exacerbating factor in some lupus patients via promoting IFN production.

## Introduction

Epstein-Barr virus (EBV) is a human gamma herpesvirus, and associated with many different human diseases including mononucleosis, systemic lupus erythematosus (SLE, also called lupus), and several other diseases [1], [2]. EBV transforms adult primary B cells into continually growing lymphoblastoid cell lines and concomitantly establishes type III latency in vitro [1]. EBV latent membrane protein-1 (LMP1) is an integral membrane protein and is required for the viral transformation process.

EBV establishes a lifelong persistent infection within peripheral blood B cells with no or extremely low LMP1 expression [3], [4]. LMP1 acts as a constitutively active, receptor-like molecule [5] and activates a variety of cellular genes that enhance cell survival, adhesive, invasive, and angiogenic potential. Remarkably, we have found that LMP1 is an antiviral gene and primes cells for type I interferon (IFN) production [6], [7].

Lupus is a chronic, systemic, autoimmune disease that affects about 0.1% of the US population. EBV has been linked to lupus pathogenesis: EBV primary infection may be associated with the onset of lupus in some patients [8], [9], [10]; certain EBV epitopes are similar to auto-antigens presented in lupus patients [11], [12]; and higher EBV viral load, EBV antibodies, EBV seroconversion rates, and EBV-infected B cells have been observed in lupus patients [13], [14]. The control of latent EBV infection is less effective in lupus patients [15], [16]. The expression of LMP1 RNA has been shown to be associated with lupus [17] and LMP1 promotes autoimmunity in certain rodent backgrounds [18].

Toll-like receptors (TLRs) are a family of evolutionarily conserved receptors that recognize molecular patterns unique to pathogens and activate host innate and adaptive immunity against pathogens [19], [20]. One of the major products from TLR activation is the production of IFNs, key components to mount a proper and robust immune response to a viral infection [21], [22]. TLRs play critical roles in lupus pathogenesis. TLR7 is associated with lupus progression [23], [24], [25], [26]. In addition, the downstream signaling components of TLR7, IFN regulatory factor 5 (IRF5) and IRF7, are closely associated with lupus pathogenesis [27], [28], [29], [30], [31], [32]. Recognition of self-nucleic acids by TLR7 and TLR9 on plasmacytoid dendritic cells is considered to be a key steps in IFN production in lupus and correlated with the severity of disease [33], [34]. Prolonged TLR3 may lead to autoimmune reaction and aggravates lupus pathogenesis [35], [36].

Type I IFNs are apparently a hallmark in lupus. IFN levels and IFN-stimulated genes (ISG), collectively called IFN signatures in some of the literature, are elevated in lupus patients [37], [38], [39]. The use of IFNs for the treatment of other diseases has caused lupus-like syndromes [40], [41]. Mice have failed to develop lupus manifestations if the IFN receptor is deleted [42]. In addition, IFN promotes survival and differentiation of mature lymphocyhtes, class switching at immunoglobulin heavy chain loci, and activation of dendritic cells [43]. IFN also enhances the activation of B lymphocytes by RNA-associated auto-antigens [44]. Therefore, the IFN pathway has emerged as a focal point for understanding mechanisms of autoimmunity in lupus.

We suspect LMP1 may contribute to lupus pathogenesis by priming cells for IFN production, and have examined the relation among LMP1, TLRs, and IFNs. We find TLR7 activation increases LMP1 expression in EBV-infected cells, and potentiates those cells for production of IFNs by TLR3 or TLR9 activation. In addition, LMP1 and IFNs are co-expressed in the same cells in some lupus patients. These results suggest EBV might be an exacerbating factor in some lupus patients by responding to aberrant TLR7 activation and promoting IFN production.

## Results

### TLR7 Stimulates the Expression of EBV LMP1

**Figure 1:**
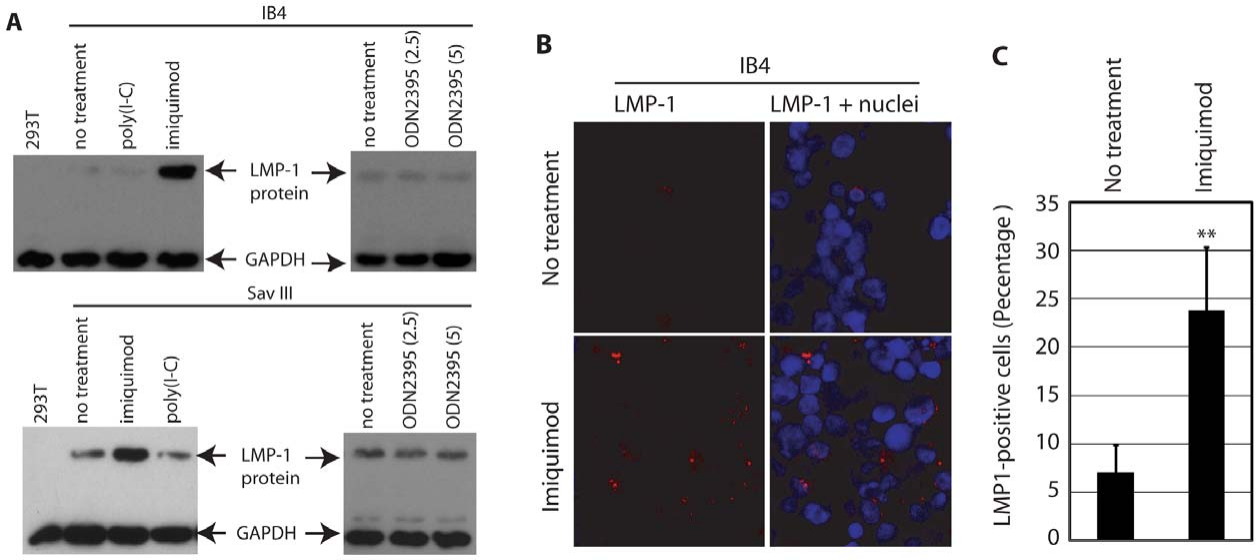
TLR7 activation stimulates the expression of EBV LMP1. A. TLR7 agonist stimulates EBV LMP1. SavIII or IB4 cells were treated with TLR7 agonist (imiquimod; 25 µg/ml), TLR3 agonist (poly (I:C); 10 µg/ml) or TLR9 agonist (ODN2395; 2.5 or 5 µM) for 24 hours. Cell lysates were used for Western blot analysis with LMP1 and GAPDH antibodies. The identity of proteins is as shown. B. TLR7 activation increases detectable LMP1-postive cells. IB4 cells were treated with imiquimod (25 µg/ml), for 24 hours, and the cells were fixed and stained with LMP1 primary and Alexa Fluor 647-labeled secondary antibodies. DAPI was used to stain the nuclei. The images were captured under identical conditions. The colors were artificially mounted to facilitate viewing. Blue, nuclei; red, LMP1. C. Quantification of detectable LMP1-positive cells. IB4 cells were treated and stained as in Panel B. The percentages of LMP1-positive cells were counted in 10 randomly selected fields. For untreated controls, the average number of cells per field with standard deviation is 201.5±59.5; while in imiquimod treated cells, the average number of cells per field with standard deviation is 170.6±70.8. The difference is statistically significant (*p*<0.01). The *p* value was calculated by paired Student's t test with the use of Microsoft Excel.

Because the LMP1 promoter region has a putative IRF responsive element [45], [46], TLRs have potential to activate IRFs, and EBV-infected cells expressing TLRs, we reasoned that LMP1 might be regulated by TLR signals in EBV-infected cells. SavIII and IB4 are EBV-transformed B cell lines with type III latency. Cells were treated with various TLR agonists and 24 hours later, Western blots were used to detect the expression of LMP1. As shown in Figure 1, TLR7 agonist (imiquimod) induced expression of LMP1 protein in two different EBV-infected cell lines. However, TLR3 and -9 agonists did not induce the expression of LMP1. TLR3, -7 and -9 are expressed in both cell lines (Figure S1), and the TLR3 and 9 agonists were both effectiveness (data not shown). Of note, IB4 is considered as prototype of EBV-transformed cells in vitro and widely used in the research about EBV transformation [47], [48], [49], [50], [51], [52]. In addition, imiquimod induced the expression of LMP1 RNA in both SavIII and IB4 cells (Figure S1, data not shown).

It is known that LMP1 expression in EBV-infected cells exhibits 100-fold differences [53]. We therefore examined whether TLR7 activation increase the percentage of cells with high LMP1 expressions. Cells were treated with imiquimod and immune stained with LMP1 antibody the next day. As shown in Figure 1B, only a small proportion of cells were positive for LMP1 in EBV-transformed IB4 cells. However under the exact same conditions, the number of LMP1-positive cells was increased drastically upon TLR7 activation and the differences are statistically significant (Figure 1C). Similar results were also obtained in SavIII cells (Figure S2). Therefore, TLR7 activation increases the population of high LMP1 expressing cells in EBV-infected cells. Collectively, all data suggest that TLR7 activation stimulates the expression of EBV LMP1 in EBV-infected cells.

### IRF7 is Involved in the TLR7-mediated LMP1 Induction

IRF7 is involved in the signaling of TLR7 [28], [54]. Interestingly, IRF7 was first discovered and highly expressed in EBV-transformed cells and is a positive regulator for LMP1 [45], [55]. It is possible that IRF7 is involved in TLR7-mediated induction of LMP1.

**Figure 2:**
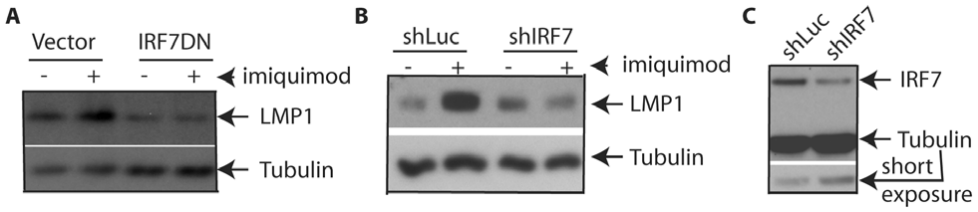
IRF7 is involved in the induction of LMP1. A. Dominant-negative mutant of IRF7 blocks the induction of LMP1. IB4 cells were transfected with pcDNA3, or IRF7DN, along with CD4 expression plasmids. After 24 hours, the transfected cells were equally split into two flasks and one was treated with imiquimod overnight (10 µg/ml). Transfected cells were enriched by the use of CD-4 positive selection kit. Cell lysates from were used for Western blot analysis with LMP1 and tubulin antibodies. The identity of proteins is as shown. B. Reduction of IRF7 affects the induction of LMP1. IB4 cells were transfected with shluc, or shIRF7 (mixture of IRF71, 72, and 73 at 1∶1∶1 ratio). The transfected cells were equally split into two flasks for imiquimod treatment overnight and were enriched. Cell lysates were used for Western blot analysis with LMP1 and tubulin antibodies. The reduction of IRF7 by shIRF7 is also shown. The images in the same box indicate that they are derived from the same membranes. The identity of proteins is as shown.

To address the role of IRF7 in LMP1 induction, IRF-7-dominant negative mutant (IRF7DN) was used [56]. A vector, or IRF7DN and a CD4 expression plasmid were transfected into IB4 cells. Cells were split into two flasks, one of which was treated by imiquimod. Transfected cells were enriched by the use of magnetic beads for CD4 expressing cells [7], [57], [58]. As shown in Figure 2A, TLR7 activation caused an increase in LMP1 protein expression in vector-transfected IB4 cells as expected; however, IRF7-DN did not enhance the expression (Figure 2A).

Similarly, we reduced the expression of IRF7 by small hairpin RNA (shRNA) technology. Several shRNA for IRF7 plasmids were transfected with CD4 expression plasmids, and the transfected cells were enriched and examined. The induction of LMP1 was blocked when IRF7 expression was reduced (Figure 2B). Therefore, IRF7 is involved in the TLR7-mediated induction of LMP1.

### EBV Lytic Replication Plays a Limited Role in the Induction of LMP1

It has been reported that TLR7 activates Kaposi's sarcoma-associated herpesvirus, a close relative to EBV, lytic replication from latently infected cells [59]. In addition, EBV LMP1 is expressed during EBV lytic replication process [60]. Therefore, it is possible that TLR7 activation leads to EBV lytic replication and furthermore increases the expression of LMP1 indirectly.

**Figure 3:**
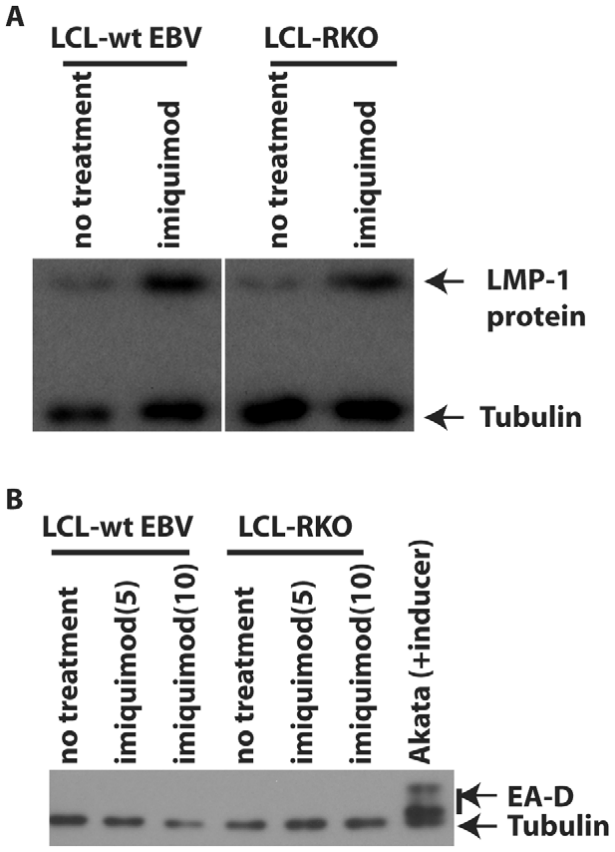
EBV lytic replication plays limited role in the induction of LMP1. A. TLR7 activation induces LMP1 in EBV-transformed cells defective for viral lytic replication. An EBV-BRLF-null virus (RKO) transformed primary B cell line (LCL-RKO) and its parental virus transformed cells (LCL-wtEBV) were treated with imiquimod overnight. Cell lysates were used for Western blot analysis with LMP1 and tubulin antibodies. The identity of proteins is as shown. B. TLR7 activation failed to induce EBV lytic replication in EBV-transformed cells. LCL-wtEBV and LCL-RKO cells were treated with imiquimod overnight. The positive control was Akata cells treated with anti-human IgG. Cell lysates were used for Western blot analysis with LMP1 and Tubulin antibodies. The identity of proteins is as shown.

To address the role of lytic replication in LMP1 induction, we examined whether TLR7 activation leads to the induction of EBV lytic replication. The expression of EBV EA-D (BMRF-1) was used as a marker for lytic replication. The essential function of EA-D in EBV lytic replication has been well established and EA-D as an indicator of lytic replication has been widely used in the field [61], [62]. Because imiquimod did not obviously induce the expression of EA-D in IB4 and Sav III cells (Figure S3), data suggest that TLR7 activation might not induce EBV lytic replication. Because, with our data, we cannot completely rule out the involvement of low levels of lytic replication in LMP1 induction, we used BRLF1-knockout EBV (EBV-RKO) transformed cells [63]. Viral lytic replication cannot be completed in EBV-RKO transformed cells because BRLF1 is required for EBV lytic replication [64]. EBV-RKO-transformed (LCL-RKO) and the corresponding wild-type virus-transformed B lymphocytes (LCL-wtEBV) were treated with TLR7 agonist. As shown in Figure 3, imiquimod did induce the expression of LMP1 in both wild type and RKO transformed cell lines. Furthermore, we observed no evidence that lytic replication was induced in these lines. Multiple bands for EA-D are a common phenomenon due to phosphorylation (Figure 3B). Therefore, data in Figure 3 and Figure S3 strongly suggested that viral lytic replication is not involved in LMP1 induction by TLR7.

### TLR7 Potentiates EBV-infected Cells for IFN Production

**Figure 4:**
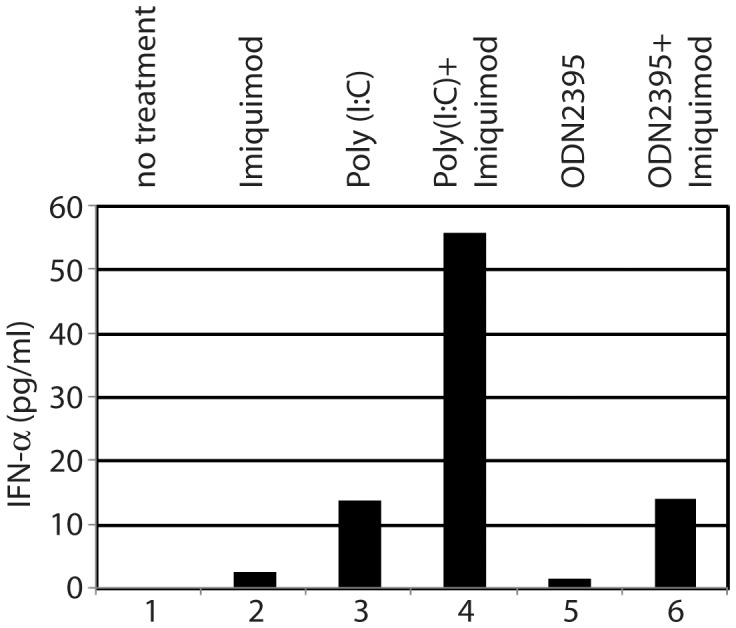
TLR7 potentiates EBV-infected cells for IFN productions. IB4 cells were treated with TLR7 agonist (imiquimod; 5 µg/ml) for 12 hours, and the cells were then treated with TLR3 (poly (I:C); 10 µg/ml) or TLR9 agonist (ODN2395; 5 µM) for 24 hours. Medium was collected for ELISA. Average of duplicates is as shown. One representative from three independent experiments is as shown.

Because B lymphocytes express many TLR molecules, we tested whether TLR agonist could activate IFN in EBV transformed cells. IB4 cells were treated with various TLR agonists and ELISA was used for the detection of multiple IFN-alpha subtypes in the culture media. As shown in Figure 4, only TLR3 activation leads to IFN production. TLR7 and 9 agonists could not (or could only very marginally) induce IFNs (lanes 2, 3, and 5).

Because TLR7 activation stimulates the expression of LMP1, we suspect that TLR7 is capable of priming cells for IFN production via LMP1. IB4 cells were treated with TLR7 agonist for 12 hours and the cells were further treated with TLR3 and -9 agonists for 24 hours. As shown in Figure 4, when cells were treated with TLR3 and -9 agonists after TLR7 stimulations, both produced more IFNs than those treated alone (lanes 4 and 6). The combination of TLR3 and 9 agonists did not result in a synergistic effect and the infection by Sendai virus resulted in much higher levels of IFNs (data not shown). Sendai virus is a common IFN inducer and can be used as a positive control. Therefore, the data in Figure 4 suggest that TLR7 activation potentiates EBV-infected cells for type I IFN production by TLR3 and -9 activations.

### EBV LMP1 and IFN are Expressed in the Same Cells in Lupus Patients

**Figure 5:**
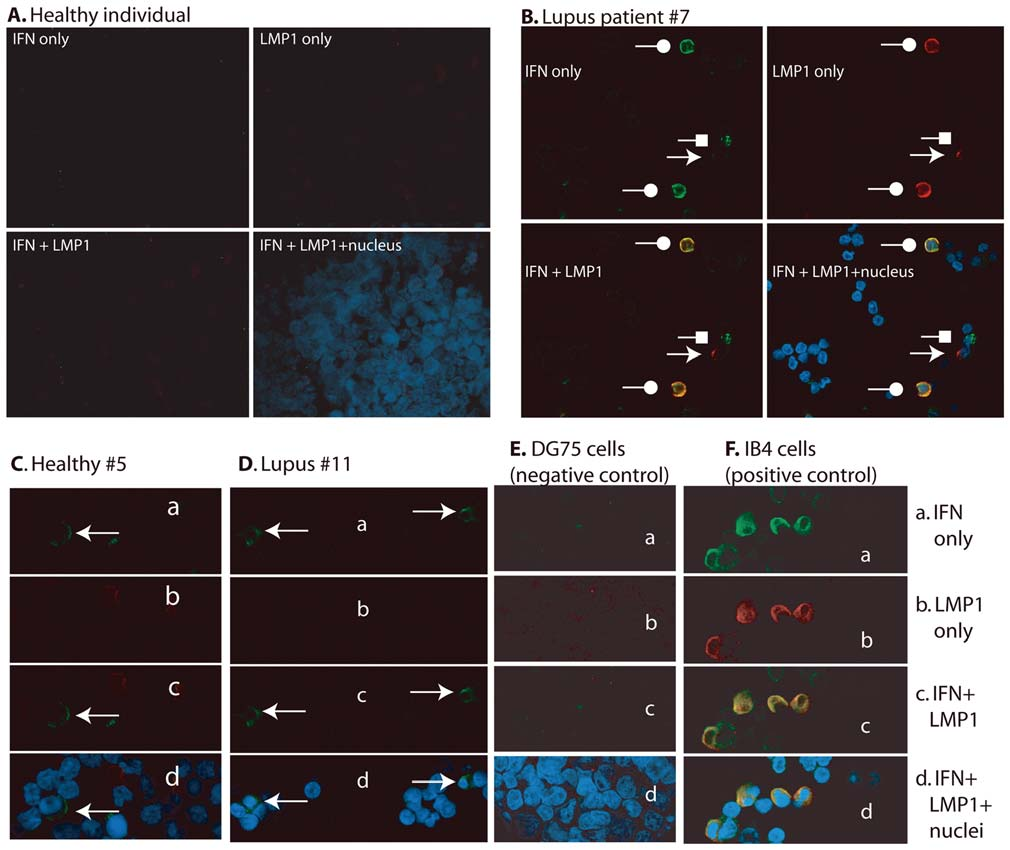
EBV LMP1 is localized in the IFN-producing cells in lupus patients. PBMCs from healthy or lupus patients were stained with IFN and LMP1 antibodies. Alexa Fluor 488- and Alexa Fluor 647-labeled secondary antibodies were used to distinguish the signals from IFN and LMP1, respectively. DAPI was used to stain the nuclei. The colors were artificially mounted to facilitate viewing: blue, nuclei; green, IFN; red, LMP1. Panel A: A representative result for apparent healthy individual is shown. Panel B: Result for lupus patients (Patient #7 in Table 1) is shown. Circle: dual-positive cells. Arrow: LMP1 single positive cell. Square: IFN- single positive cell. Panel C: Healthy individual #5. Arrow indicates an IFN-positive cell. Some red blood cells are presents (no nuclei); Panel D: Result for lupus patients #11: arrows indicate the IFN-positive cells; Panel E: DG75 cells (negative control); Panel F: IB4 cells infected with Sendai virus (positive control). Panels: C–F: a, IFN only; b, LMP1 only; c, IFN plus LMP1; and d, IFN, LMP1, and nuclei.

Because TLR7 is associated with lupus, we suspect that EBV may contribute to IFN production in lupus patients. We examined 20 lupus patients' peripheral blood mononuclear cells (PBMC) by immunocytochemistry analyses. Patients' PBMCs were immediately fixed and processed for immunostaining for both IFN and LMP1 expression. Because collection of different patients' blood took a long period of time, half of patients' PBMCs were stored. Once all 20 patients' blood was collected, we thawed the stored PBMCs and processed them simultaneously for all samples. The specificity of the IFN-alpha Ab was confirmed Figure S4). DG75 (EBV-negative) and Sendai virus infected IB4 cells (EBV-positive) were used as negative and positive controls, respectively. These controls were used to set the proper settings for confocal microscopy (Figures 5E, 5F). All samples were examined with the same settings on the same machine.

With this assay, LMP1 was not detected in all six healthy individuals (Table 1). Interestingly, we had detected that two out of six healthy individuals (33%) had IFN-positive cells (Figure 1 and Table 1). The results may be related to the fact that IFNs could be induced by many factors, including some physiological stresses. Also, these individuals were “apparently healthy” at the time of blood collection. All lupus patients had detectable IFN-positive cells, and the majority of them were also LMP1-positive (16 out of 20; 80%). The co-expression of IFN and LMP1 were detected in the majority of patients (12/20; 60%) (Figure 5; Table 1). Therefore, both IFN and LMP1 are highly expressed in lupus patients; furthermore, IFN and LMP1 are co-expressed in the same cells in some lupus patients.

**Table 1:** Detection of cellular IFN and viral LMP1 in lupus patients.

Patient #	SELENA SLEDAI	IFN	LMP1	Co-expression in same cells	Descriptor
**1**	8	**+**	**+**	**+**	Arthritis (4), Pleurisy (2), Increased DNA Binding (2)
**2**	6	**+**	**+**	**+**	Arthritis (4) Increased DNA Binding (2)
**3**	8	**+**	**+**	**−**	Arthritis (4) Low Complement (2) Increased DNA Binding (2)
**4**	8	**+**	**−**	**−**	Arthritis (4) Low Complement (2) Increased DNA Binding (2)
**5**	6	**+**	**+**	**+**	Arthritis (4) Rash (2)
**6**	10	**+**	**+**	**+**	Arthritis (4) Rash (2) Low Complement (2) Increased DNA Binding (2)
**7**	14	**+**	**+**	**+**	Arthritis (4) Myositis (4) Rash (2) Low Complement (2) Increased DNA Binding (2)
**8**	2	**+**	**+**	**+**	Low Complement (2)
**9**	8	**+**	**+**	**+**	Arthritis (4) Low Complement (2) Increased DNA Binding (2)
**10**	6	**+**	**+**	**−**	Arthritis (4) Low Complement (2)
**11**	6	**+**	**−**	**−**	Arthritis (4) Low Complement (2)
**12**	12	**+**	**+**	**+**	Arthritis (4) Proteinuria (4) Rash (2) Increased DNA Binding (2)
**13**	6	**+**	**+**	**+**	Arthritis (4) Low Complement (2)
**14**	9	**+**	**−**	**−**	Arthritis (4) Low Complement (2) Increased DNA Binding (2) Leukopenia (1)
**15**	9	**+**	**−**	**−**	Arthritis (4) Low Complement (2) Increased DNA Binding (2) Leukopenia (1)
**16**	6	**+**	**+**	**+**	Arthritis (4) Thrombocytopenia (1) Leukopenia (1)
**17**	5	**+**	**+**	**−**	Arthritis (4) Leukopenia (1)
**18**	5	**+**	**+**	**+**	Arthritis (4) Leukopenia (1)
**19**	8	**+**	**+**	**−**	Proteinuria (4) Rash (2) Increased DNA Binding (2)
**20**	8	**+**	**+**	**+**	Arthritis (4) Rash (2) Increased DNA Binding (2)
**N1**	N/A	**+**	**−**	**−**	N/A
**N2**	N/A	**−**	**−**	**−**	N/A
**N3**	N/A	**−**	**−**	**−**	N/A
**N4**	N/A	**−**	**−**	**−**	N/A
**N5**	N/A	**+**	**−**	**−**	N/A
**N6**	N/A	**−**	**−**	**−**	N/A

The SELENA-SLEDAI scores and the various descriptors at the time of blood collections are listed. The positive cells were identified by expression intensity similar to the positive control (Figure 5E). The numbers of cells for either IFN-alpha or LMP1-postive are very few, and the percentages could not be used to represent the frequency. If several positive cells were positively identified in a specimen, the score “+” was given. However if two or fewer positive cells were identified in approximate 5×10^6^ PBMC, it were scored as negative (−). The reason to use two positive cells as a cutoff was to avoid any artificial signals. N1–N6: normal, healthy individuals' bloods.

## Discussion

It is known that primary EBV infection leads to TLR7 inductions [65]. In this report, we provide evidence that TLRs regulate EBV gene expression. First, TLR7 stimulates the expression of LMP1 and increases the population of high-LMP1 expression cells (Figure 1), and the increase in LMP1 expression is apparently at the RNA level (Figure S1). Second, TLR7 activates IRF7 and EBV-latency cells express high levels of IRF7. We show that IRF7 is involved in the TLR7-mediated induction of LMP1 (Figure 2). Third, because LMP1 was induced in a lytic-replication-defective EBV-transformed B lymphocytes (LCL-RKO) (Figure 3; Figure S3), the induction of LMP1 is apparently not related to the EBV lytic replication process. We also tested whether LMP1 could be increased further by TLR7 activation in induced Akata cells. The results were not clear (data not shown). We suspect that because lytic replication already induces the expression of LMP1, the TLR7 activation may not be very effective at that stage. In addition, Akata is a Burkitts' lymphoma line that already has some genomic mutations. In summary, compelling evidence suggests that TLR7 regulates EBV LMP1 expression. Finally, it is known that LMP1 primes cells for IFN production, and we find TLR7 actually primes EBV-infected cells for IFN production induced by TLR3 and TLR9 (Figure 4). Because LMP1 is required for the growth of the EBV-transformed cells, ablation of LMP1 in EBV-transformed cells would have a strong adverse impact on cellular growth; thus, the effects on IFN production in the ablation of LMP1 would be hard to observe and interpret. Therefore, our data simply suggest the association of LMP1 with the synergy.

To examine whether the TLR7-LMP1 relation is operative in vivo, we have examined PBMC from lupus patients because TLR7 activation is associated with lupus pathogenesis. We provide evidence that EBV LMP1 protein is highly expressed in lupus patients (Figure 5 and Table 1), confirming a previous report using a different technique [17]. Because aberrant TLR7 activation is associated with lupus pathogenesis, high LMP1 expression in lupus might be related to the fact that TLR7 stimulates LMP1 expression in tissue cultured cells (Figure 1, 2, Figure S1).

Lupus patients have high IFNs or IFN signatures in PBMCs. High levels of LMP1 may explain a high IFN signature in lupus: LMP1 induces ISGs [7], [66]. Our results therefore suggest a novel mechanism for TLR activation to increase ISGs expression in lupus patients. Furthermore, we find that LMP1 and IFNs are co-expressed in the same cells in lupus' PBMCs (Figure 5 and Table 1). Although we do not know the identity of EBV-infected cells in lupus at this time, the current data suggest that EBV-infected cells are likely to be a source of IFNs in lupus patients.

**Figure 6:**
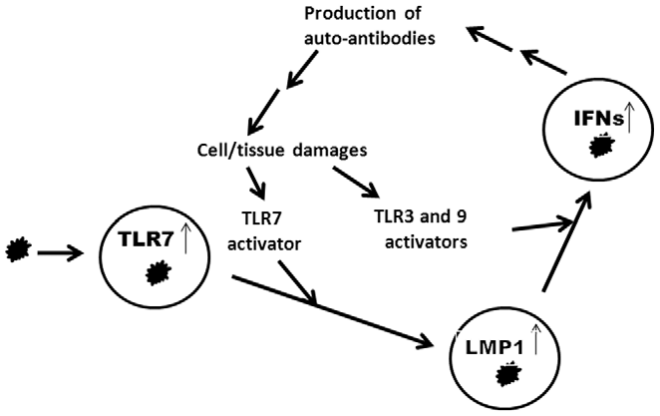
Potential exacerbating role of EBV in lupus pathogenesis via IFNs. EBV infection increases the expression of TLR7. EBV-infected cells may respond to TLR7 stimulators present in lupus patients, and increase the expression levels of LMP1; LMP1 primes the cells for IFN production; TLR3 and TLR9 stimulators induce IFN production in those EBV-infected cells. High amounts of IFNs would be responsible for more auto-antibody productions, cell/tissue damages, and eventually more self-nucleotide complexes containing various TLR activators, including for TLR7; TLR7 activation again may promote more LMP1 expression.

The linkage between TLR7 and LMP1 expression is intriguing: primary infection of B lymphocytes by EBV may induce the expression of TLR7, IRF5, and IRF7 [55], [65], [67], [68], [69]. Further, the type of viral latency cells with high TLR7, IRF-5, and IRF7 (type III) are probably present in lupus patients in vivo [12], [70]. Because the type III latency cells are resistant to IFN-mediated growth inhibition [71], [72], those EBV-infected cells may be preferentially propagated in lupus patients. TLR7 and -9 activations are associated with lupus, and the TLR7 and -9 dual antagonists alleviate lupus pathogenesis [25], [26], [73], [74]. In addition, high LMP1 expression is associated with lupus severity [17]. In our experimental system, TLR7 itself hardly induces IFNs by B lymphocytes, but potentiates IFN production by TLR3 or TLR9 agonists in EBV-infected cells (Figure 4). Therefore, our data correlate well with the known roles of TLR7and -9 in lupus pathogenesis, and suggest an exacerbating cycle in lupus patients: EBV infection induces TLR7 expression; TLR7 activation promotes LMP1 expression; LMP1 might potentiate the cells for IFN production by TLR3 and TLR9 agonists; and high amounts of IFNs would promote more auto-antibody productions, cell/tissue damages, and eventually more self-nucleotide complexes to activate TLRs [75], [76], [77], [78], which may again promote LMP1 expression (Figure 6). Because some case reports suggest that primary EBV infection is associated with the onset of lupus [8], [9], [10], it is tempting to speculate that EBV may even play an etiological role in some lupus-susceptible individuals as both an initiator and an exacerbating factor in IFN productions and furthermore the development of the diseases.

LMP1 is a primary oncoprotein for viral transformation [2]. Our results suggest that lupus patients may have high incidence of EBV-associated lymphomas. Actually, lupus patients exhibit three- to four-fold increase in the risk of developing B cell malignancies [79], [80], and EBV may be associated with some lymphomas [81], [82]. In addition, EBV is a factor and associated with Hodgkin's and non-Hodgkin' lymphomas in AIDS patients. Because HIV activates TLR7 pathway [83], the linkage between TLR7 and EBV might play a role in the development of AIDS-associated lymphomas.

In summary, we have provided evidence that TLR7 activation for the enhanced LMP1 expression and this mechanism may be present in lupus in vivo. These data may support EBV as an exacerbating agent in the development of lupus through modulation of IFNs.

## Materials and Methods

### Cell Culture, Plasmids, Sendai Virus, and Western Blot Analysis

DG75 and Akata are EBV-negative and EBV-positive Burkitts' lymphoma cell lines, respectively [84]. SavIII, IB4, LCL-wtEBV, and LCL-RKO are all EBV-transformed cell lines in vitro [48], [63], [85]. LCL-wtEBV, and LCL-RKO were obtained as gifts from Dr. Shannon Kenney [48], [63], [85]. These cells were maintained in RPMI-1640 plus 10% fetal bovine serum (FBS; Gibco BRL) and 1% Penicillin-streptomycin (PS) at 37°C in 5% CO_2_ incubation. Sendai virus stock was purchased from Spafas, Inc. For Sendai infection, 5–10 HA units/ml Sendai virus were added to the target cells for 12 hours. Cells then were fixed for further analysis. 293T is a human fibroblast line (from ATCC) and were grown in Dulbecco's modified Eagle medium (DMEM, Gibco BRL) supplemented with 10% FBS. 293T cells were seeded and grown to 40–50% confluence in eight-chamber culture slides (BD Falcon; 354108). Effectene was used for transfection according to the manufacturer's instructions (Qiagen, Inc). After 24 hours, media were collected, and the cells were used for immunostaining. The expression plasmid for human IFN-α2 is a gift from Dr. Blake Roessler [86]. Human IFN-beta expression plasmid was purchased from OriGene (SC127861). IRF7 dominant negative mutant (IRF7-DN) and CD4 expression plasmids are described previously [56], [67], [87]. Western blot analysis was essentially the same as described previously [55], [88], [89]. shLuc was also described previously [90]. shIRF7 was the mixture of shIRF71, -72, and -73 in a 1∶1∶1 ratio. The shIRF71(target sequence (5′-TGGCCCGCCCCCCGAGGCT-3′), shIRF72 (5′-AATGGCCTTGGCTCCTGAG-3′), and shIRF73 (5′-GCCCGCGGCAGGTGGCCGC-3′) were all cloned into the pHP vector, an shRNA expression plasmid [46]. The target sequences were confirmed by sequencing analysis. LMP1 antibody (CS1-4) was purchased from Dako. GAPDH antibody was obtained from Santa Cruz Biotechnology Inc (sc-47724). Tubulin (T6557) and FLAG antibody (F1804) were obtained from Sigma.

### Reverse Transcriptase (RT)-Polymerase Chain Reaction (RT-PCR)

RNA was extracted from cells using the TRIzol method, and was synthesized into cDNA using Superscript II RT from Invitrogen. The cDNA was then used in a PCR reaction with primers for LMP1 and Actin. Primer sequences for LMP1 were 5′– CCCAAGCTTTTAGTCATAGTAGCTTAGCTG – 3′ and 5′ – CGGGATCCGGAGGTGGCGGTGGACATGGACCACGACACACTGATGAACACCACCAC – 3′. The primers for Actin were 5′ – TTCTACAATGAGCTGCGTGT – 3′ and 5′ – GCCAGACAGCACTGTGTTGG – 3′. Primer sequences for TLR3 were 5′-GCATTTGTTTTCTCACTCTTT -3′and 5′-TTAGCCACTGAAAAGAAAAAT-3′ Primer sequences for TLR7 were 5′-AAACTCCTTGGGGCTAGATG -3′and 5′-AGGGTGAGGTTCGTGGTGTT-3′. Primer sequences for TLR9 were 5′-CGCCCTGCACCCGCTGTCTCT-3′ and 5′-CGGGGTGCTGCCATGGAGAAG-3′.

### Transfection and Enrichment of Transfected Cells

Electroporation (300 V; 975 microfarads) was used for transfection of the IB4 cells with total 5 µg of DNA including 1 µg of CD4-expression plasmids. One or two days after transfection, the cells were treated with or without imiquimod (5–10 µg/ml) for 24 hours. Enrichment for CD-4-positive cells was performed with the use of anti-CD-4-antibody conjugated to magnetic beads according to the manufacturer's recommendation (Dynal, Inc.).

### TLR Treatment and IFN-α Measurement

TLR agonists, poly(I:C) (tlrl-pic) for TLR3 stimulation (10 µg/ml), imiquimod (tlrl-img) for TLR7 stimulation (5–10 µg/ml), and ODN 2395(tlrl-2395) for TLR9 stimulation (2–5 µM) were purchased from Invivogen. In addition, imiquimod (IMG-2207-1) was also obtained from Imgenex. While different batches and sources of imiquimod often gave similar results, the batch of FBS may influence the outcomes of the treatments. For TLR7 potentiating experiments, imiquimod was used to treat cells for 12 hours and then cells were treated with other reagents. The supernatants were used for IFN measurements. The concentration of IFN-α was determined by a commercially available human interferon alpha (Hu-IFN-alpha) ELISA kit (PBL Biomedical Laboratories; catalog number 41100) according to the manufacturer's recommendations. The kit is capable of detecting several human IFN-alpha subtypes, but not IFN-beta. Samples were measured in duplicates.

### Ethics Statement

The research was approved by the Institutional Review Board (IRB) of the University of Nebraska-Lincoln.

### Patients' and Healthy Individuals' Bloods

After informed consent, whole blood samples from 20 lupus patients were collected at the Arthritis Center of Nebraska and immediately sent to The University of Nebraska-Lincoln. The Systemic Lupus Erythematosus Disease Activity Index (SLEDAI) scale, as modified by the Safety of Estrogen in Lupus Erythematosus National Assessment (SELENA) scores, were calculated following standard procedures on each patient at the time of blood draw [91], [92], [93]. Heparin was used as the anticoagulant. The fresh whole bloods from healthy individuals were purchased from Zen-Bio, Inc (SER-WB10ML). These bloods were processed immediately upon on arrival.

### Isolation of Peripheral Blood Mononuclear Cells (PBMC)

Blood was diluted with 1× Phosphate Buffered Saline (PBS). PBMCs were isolated from blood with the use of Ficoll-Paque™ PLUS following manufacturer's recommendations (GE Healthcare). PBMC were collected and counted and some are directly for the fixation process, others suspended in 10% DMSO and 90% FBS and stored in a liquid nitrogen tank for future analysis.

### Immunocytochemistry Analysis

Cells were aliquoted into 1.5 mL centrifuge tubes, and spun down for 1 minute at 4,000 rpm in a microcentrifuge. Cells were washed with 1XPBS for 5 minutes and then fixed with 1 mL 4% Paraformaldehyde for 15 minutes. Cells were then rinsed twice in PBS, permeabilized with 95% cold methanol at −20°C for 5 minutes, and allowed to dry on poly-lysine slides. Cells on slides were washed twice with 1× PBS in a gently shaken slide container, and then blocked with PBST (1XPBS+ 0.5% Tween 20) including 3% BSA for 30 minutes. The slides were kept in the dark for the remainder of the experiment. The cells were incubated in primary antibodies with 1∶100 dilutions for LMP1 [CS1-4 (DAKO) or S-12 (BD-Pharmingen)] and 1∶50 dilution for human IFN-alpha antibody (PBL Biomedical Laboratories; 31101-1) in PBST with 1% BSA for 1.5 hours, washed with PBST, then incubated for one hour with secondary antibodies in 1∶500 in PBST with 1% BSA. The secondary antibodies were from Invitrogen (Alexa Fluor 647-Alexa Fluor® 647 goat anti-mouse IgG (H+L); A-21235 and Alexa Fluor® 488 donkey anti-rabbit IgG (H+L); A21206). Cells were washed three times, stained with 4′,6-diamidino-2-phenylindole (DAPI) for 5 minutes, and washed and mounted with Gel Mount Aqueous Mounting Medium and let air dry in the dark for at least 1 hour. Slides were kept at 4°C before examination with confocal microscopy. All samples were screened using single excitation laser line/single emission display at 405 nm/420 nm, 488 nm/522 nm, or 633 nm/660 nm, for nuclear stain, IFN or LMP1 signals, respectively. Optical images were collected under the same conditions/confocal settings for negative/positive controls and patients' samples, using the sequential scanning and simultaneous display mode of an Olympus FV500 confocal imaging system.

## Supporting Information

Figure S1 A. TLR7 agonist increase LMP1 RNA. SavIII cells were treated with TLR7 agonist (imiquimod; 25 µg/ml) for 24 hours. RNA was isolated and RT-PCR was employed to examine LMP1 RNA expression. Proper primers were used for detection of LMP1 and actin RNA respectively. PCR DNAs were separated in 8% polyacrylamide gels. The plus or minus RT for cDNA synthesis was used as a control. Input amount were shown. Size of the DNA markers is as shown on the left in base pairs (bp). The identity of target RNA is as shown. B. Expression of TLRs in EBV-transformed cells. RNA was isolated from SavIII and IB4 cells, and RT-PCR was employed to examine various TLR expression. PCR DNAs were separated in 8% polyacrylamide gels. The plus or minus RT for cDNA synthesis was used as a control. Size of the DNA markers is as shown on the left in base pairs (bp).
(TIF)

Figure S2 **Cells with high LMP1 are increased upon TLR7 activation.** SavIII cells were treated with TLR7 agonist (imiquimod; 10 µg/ml) for 12 hours, and the cells were then fixed for immunestaining experiments. LMP1 and Alexa Fluor 647-labeled secondary antibodies were used. DAPI was used to stain the nuclei. Blue, nuclei; red, LMP1. Identical settings were used to capture the images.
(TIF)

Figure S3 **TLR7 activation failed to induce EBV lytic replication in EBV-transformed cells.** IB4 and Sav III were treated with imiquimod (25 µg/ml) overnight. The positive control was Akata cells treated with anti-human IgG. Cell lysates from were used for Western blot analysis with LMP1 and Tubulin antibodies. The membrane was stripped and probed with another antibody. The images in the same box indicate that they are derived from the same membranes. The identity of proteins is as shown.
(TIF)

Figure S4 **Specificity of the IFN-α antibody.** 293T cells were transfected with expression plasmid for IFN-α2, IFN-β, or pcDNA3 (vector control) respectively. 24 hours after transfection, cells were stained with IFN-α antibody. Alexa Fluor 488-labeled secondary antibody was used to detect the expression. DAPI was used to stain the nuclei. The colors were artificially mounted to facilitate viewing. Blue, nuclei; green, IFN. The expression of IFN-β was confirmed by a functional assay (data not shown).
(TIF)
