# Supplementary figures and images for: Toll-Like Receptor 7 Stimulates the Expression of Epstein-Barr Virus Latent Membrane Protein 1
Source: PLoS One. 2012 Aug 31;7(8):e43317. doi: 10.1371/journal.pone.0043317 (PMC3432040; doi:10.1371/journal.pone.0043317)

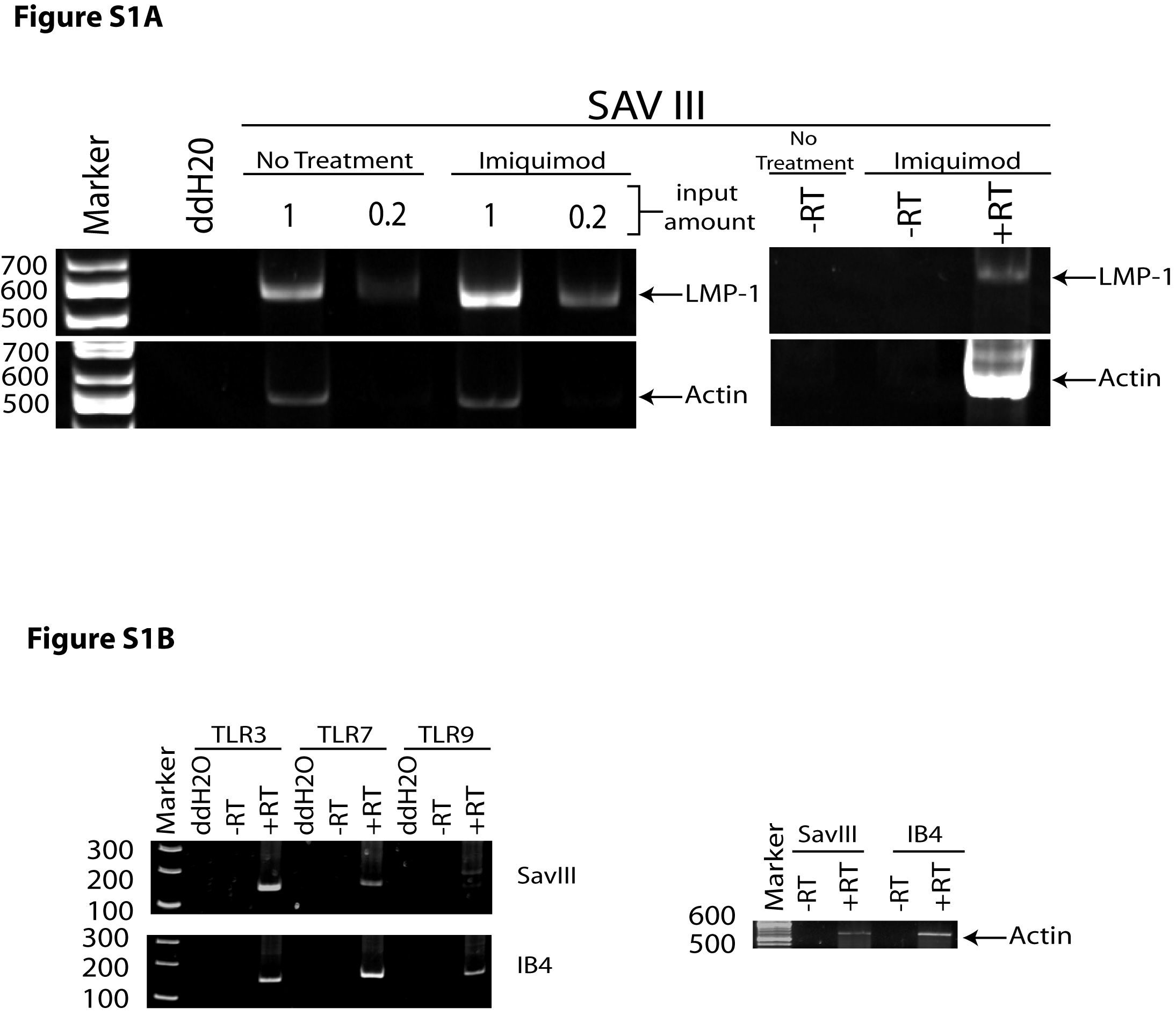

Supplement: Figure S1 — A. TLR7 agonist increase LMP1 RNA. SavIII cells were treated with TLR7 agonist (imiquimod; 25 µg/ml) for 24 hours. RNA was isolated and RT-PCR was employed to examine LMP1 RNA expression. Proper primers were used for detection of LMP1 and actin RNA respectively. PCR DNAs were separated in 8% polyacrylamide gels. The plus or minus RT for cDNA synthesis was used as a control. Input amount were shown. Size of the DNA markers is as shown on the left in base pairs (bp). The identity of target RNA is as shown. B. Expression of TLRs in EBV-transformed cells. RNA was isolated from SavIII and IB4 cells, and RT-PCR was employed to examine various TLR expression. PCR DNAs were separated in 8% polyacrylamide gels. The plus or minus RT for cDNA synthesis was used as a control. Size of the DNA markers is as shown on the left in base pairs (bp). (TIF) [file pone.0043317.s001.tif]

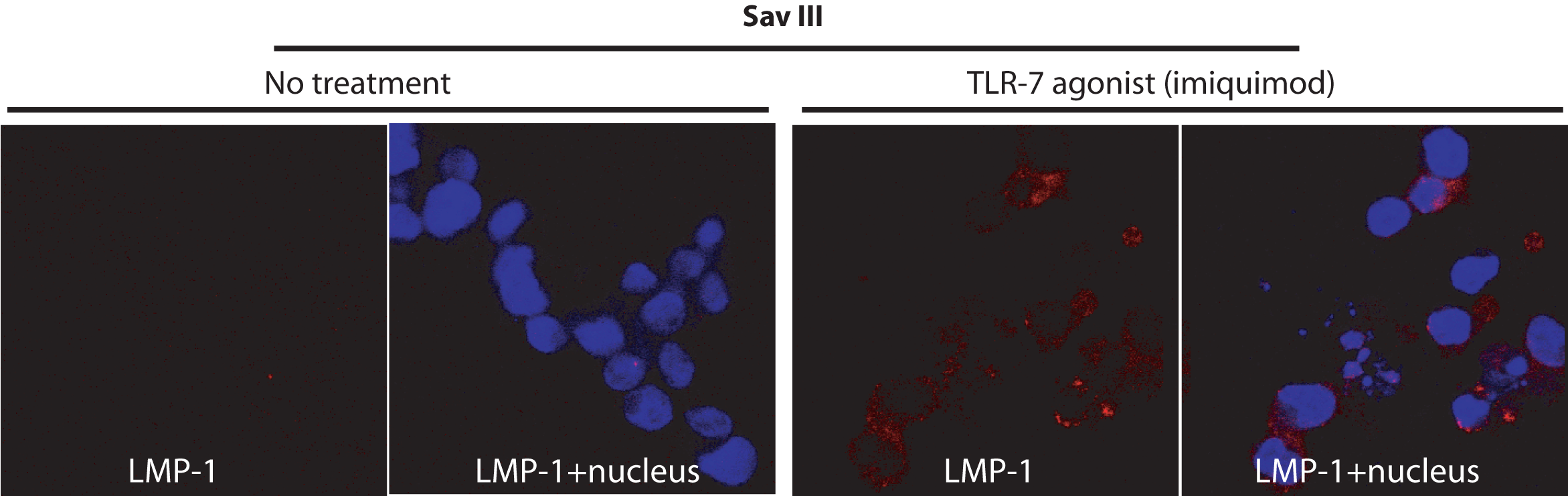

Supplement: Figure S2 — Cells with high LMP1 are increased upon TLR7 activation. SavIII cells were treated with TLR7 agonist (imiquimod; 10 µg/ml) for 12 hours, and the cells were then fixed for immunestaining experiments. LMP1 and Alexa Fluor 647-labeled secondary antibodies were used. DAPI was used to stain the nuclei. Blue, nuclei; red, LMP1. Identical settings were used to capture the images. (TIF) [file pone.0043317.s002.tif]

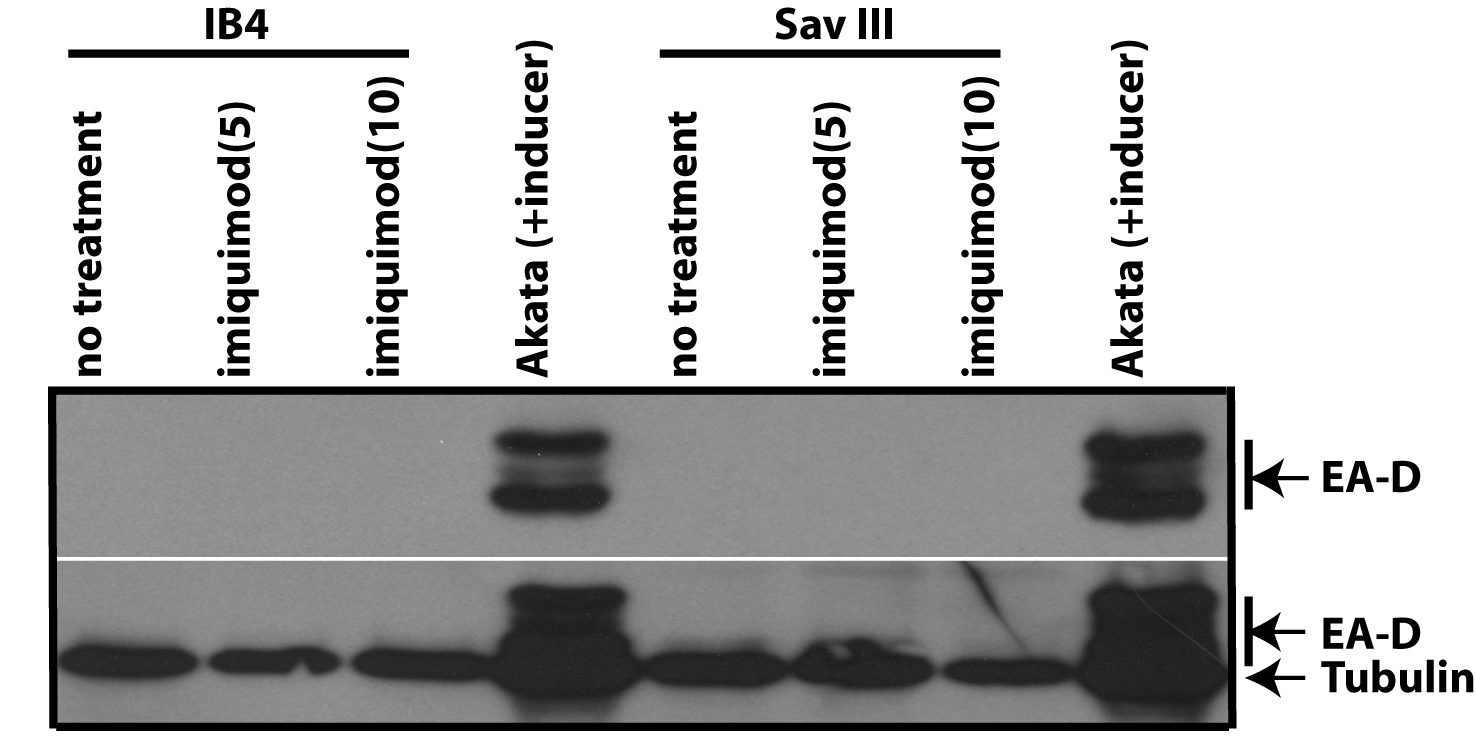

Supplement: Figure S3 — TLR7 activation failed to induce EBV lytic replication in EBV-transformed cells. IB4 and Sav III were treated with imiquimod (25 µg/ml) overnight. The positive control was Akata cells treated with anti-human IgG. Cell lysates from were used for Western blot analysis with LMP1 and Tubulin antibodies. The membrane was stripped and probed with another antibody. The images in the same box indicate that they are derived from the same membranes. The identity of proteins is as shown. (TIF) [file pone.0043317.s003.tif]

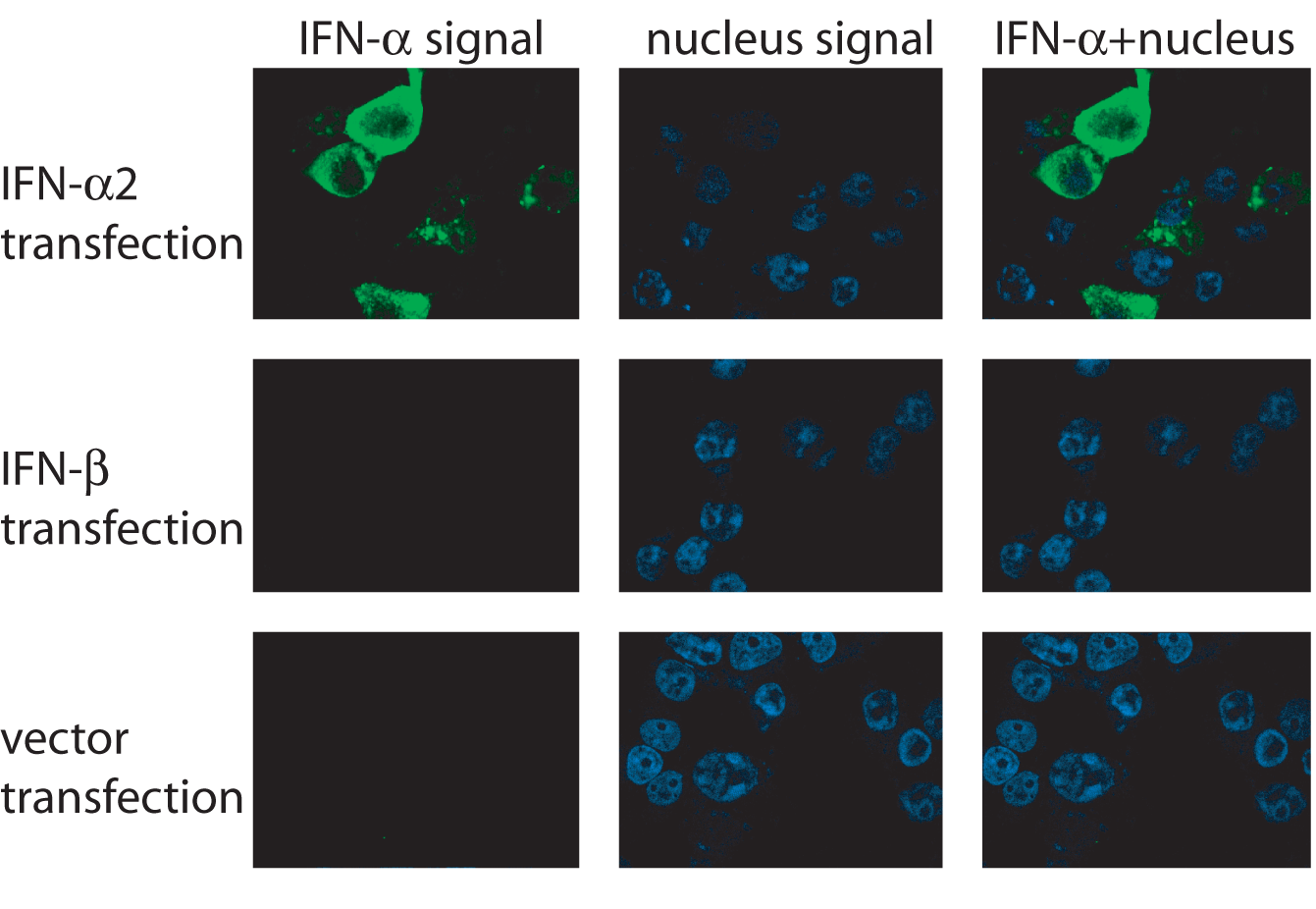

Supplement: Figure S4 — Specificity of the IFN-α antibody. 293T cells were transfected with expression plasmid for IFN-α2, IFN-β, or pcDNA3 (vector control) respectively. 24 hours after transfection, cells were stained with IFN-α antibody. Alexa Fluor 488-labeled secondary antibody was used to detect the expression. DAPI was used to stain the nuclei. The colors were artificially mounted to facilitate viewing. Blue, nuclei; green, IFN. The expression of IFN-β was confirmed by a functional assay (data not shown). (TIF) [file pone.0043317.s004.tif]
